# Diagnostic Performance of HbA1c for Detecting OGTT-Diagnosed Diabetes in Obese Individuals with Suspected Prediabetes

**DOI:** 10.3390/jcm15010374

**Published:** 2026-01-04

**Authors:** Abdullah Budak, Ihsan Solmaz, Ömer Faruk Alakuş, Bilgin Bahadır Başgöz

**Affiliations:** 1 Department of Internal Medicine, Kızıltepe District State Hospital, Mardin 47400, Türkiye; abdullahbdk635@gmail.com; 2 Department of Internal Medicine, Diyarbakır Gazi Yasargil Training and Research Hospital, Diyarbakır 21010, Türkiye; omerfaruk01@gmail.com (Ö.F.A.); bbbasgoz@gmail.com (B.B.B.)

**Keywords:** diabetes mellitus, prediabetes, obesity, HbA1c, OGTT

## Abstract

**Background:** We aimed to investigate the diagnostic performance between the oral glucose tolerance test (OGTT) and HbA1c in diagnosing prediabetes and diabetes among obese individuals, and to evaluate the diagnostic performance of HbA1c for detecting OGTT-defined diabetes in obese individuals referred for evaluation of suspected prediabetes. **Methods:** Individuals with prediabetes were included between 1 January 2020 and 31 December 2022. Participants were categorized as mildly, moderately, morbidly, or super obese based on body mass index (BMI). According to the 75 g OGTT results, patients were classified into three groups: isolated impaired fasting glucose (IFG), combined IFG + impaired glucose tolerance (IGT), and overt type 2 diabetes mellitus (T2DM). The threshold HbA1c value for T2DM diagnosis in obese patients was determined based on OGTT outcomes. **Results:** Of the 139 prediabetic obese patients included, 115 (82.7%) were female, with a mean age of 45.18 ± 11.74 years. Based on BMI, 34 patients (24.5%) were mildly obese, 41 (29.5%) moderately obese, 49 (35.3%) morbidly obese, and 15 (10.8%) super obese. According to the 75 g OGTT results, 37.4% (*n* = 52) had isolated IFG, 45.3% (*n* = 63) had combined IFG + IGT, and 17.3% (*n* = 24) had overt T2DM. A weak–moderate positive correlation was observed between HbA1c and fasting blood glucose (Spearman’s rho = 0.263, *p* = 0.002). ROC–AUC analysis showed that HbA1c had significant discriminatory power in detecting T2DM diagnosed by the 75 g OGTT (AUC = 0.881, 95% CI: 0.816–0.946, *p* < 0.001). The optimal HbA1c cut-off was 6.15%, with 83.3% sensitivity and 80% specificity. The positive predictive value was 46.1%, and the negative predictive value was 95.8%. **Conclusions:** An HbA1c threshold of 6.15% demonstrated optimal performance for detecting OGTT-defined diabetes in obese individuals with suspected prediabetes. This value should not be interpreted as a population-wide diagnostic threshold. These findings indicate that HbA1c may serve as a useful screening tool to identify obese individuals who warrant confirmatory OGTT testing, rather than as a stand-alone diagnostic criterion. Further large-scale studies are warranted to confirm these results and support future clinical guidelines.

## 1. Introduction

Obesity is a chronic disease resulting from a complex interaction of environmental, genetic, and lifestyle factors, and is associated with increased morbidity and mortality [1]. The global prevalence of obesity has nearly tripled over the past 50 years. This uncontrolled rise, in particular, has markedly increased the number of individuals with diabetes [2]. Obesity is the second leading cause of preventable deaths after smoking and is linked to numerous health complications and substantial healthcare costs. These include cardiovascular diseases, type 2 diabetes mellitus (T2DM), prediabetes, hypertension (HT), cerebrovascular diseases, sleep apnea syndrome, various cancers, non-alcoholic fatty liver disease (NAFLD), osteoarthritis, mood disorders, and infertility [3]. The diagnosis of obesity is based on the calculation of body mass index (BMI) derived from measured height and weight. BMI is defined as weight (kg) divided by height squared (m^2^) and serves as an indicator of excess body fat. Additional anthropometric measurements, such as waist and hip circumference, can also assist in the assessment of obesity [4].

T2DM is a chronic metabolic disorder characterized by hyperglycemia resulting from insulin resistance in peripheral tissues, often accompanied by a relative or absolute deficiency of insulin secretion [5]. The Global Burden of Disease 2021 analysis estimated that 529 million people were living with diabetes globally, with numbers projected to exceed 1.3 billion by 2050 [6]. Diabetes is currently the fourth leading cause of death worldwide, accounting for approximately 3 million deaths annually, and it contributes substantially to global morbidity and mortality [7].

T2DM is a lifelong disease that requires continuous care and a multidisciplinary management approach to minimize the risk of both acute and chronic complications [5]. The diagnosis of diabetes can be established using one of the following criteria: fasting plasma glucose ≥ 126 mg/dL, 2 h plasma glucose ≥ 200 mg/dL during a 75 g oral glucose tolerance test (OGTT), HbA1c ≥ 6.5%, or random plasma glucose ≥ 200 mg/dL in a patient presenting with symptoms of hyperglycemia [8].

Most T2DM patients progress through a prediabetic stage before developing overt diabetes. Insulin resistance, impaired incretin response, and compensatory hyperinsulinemia play central roles in the pathophysiology of prediabetes [6]. The diagnostic criteria for prediabetes are as follows: fasting plasma glucose 100–125 mg/dL, 2 h plasma glucose 140–199 mg/dL after a 75 g OGTT, and HbA1c 5.7–6.4% [8].

Obesity also induces several physiological changes that can influence HbA1c values independently of plasma glucose levels. Chronic low-grade inflammation, increased oxidative stress, altered iron metabolism, and a shortened erythrocyte lifespan may all affect hemoglobin glycation kinetics and lead to discrepancies between measured HbA1c values and true glycemic exposure [9]. These obesity-associated mechanisms raise concerns regarding the universal use of the 6.5% diagnostic threshold, which was originally derived from cohorts with substantially lower BMI. This biological variability further reinforces the rationale for evaluating whether alternative HbA1c cut-offs may be more accurate in obese individuals.

Previous studies have reported that the diagnostic performance and optimal cut-off values of HbA1c may vary in individuals with obesity, likely due to obesity-related alterations in erythrocyte turnover, chronic low-grade inflammation, and metabolic heterogeneity. Incani et al. demonstrated that the conventional HbA1c threshold of 6.5% showed reduced diagnostic sensitivity in obese adults, suggesting that excess adiposity may modify HbA1c–glucose relationships [10]. Similarly, Mellergård et al. found that higher BMI was independently associated with greater HbA1c variability, indicating that a single uniform threshold may not accurately reflect glycemic status across different levels of adiposity [11]. These findings support the need to reassess the applicability of current HbA1c thresholds in obese populations.

In this study, we aimed to evaluate the diagnostic performance between OGTT and HbA1c in diagnosing prediabetes and T2DM among individuals with obesity, and to determine whether a lower HbA1c threshold could improve diagnostic accuracy in this population. Since current diagnostic cut-offs are largely derived from non-obese cohorts, it remains uncertain whether the standard 6.5% threshold is optimal for diabetes detection in obese individuals. Addressing this gap was the primary motivation for the present study.

## 2. Materials and Methods

### 2.1. Study Overview

This retrospective single-center study was designed and reported as a diagnostic accuracy study in accordance with the STARD 2015 guidelines. This study was conducted at SBÜ Diyarbakır Gazi Yaşargil Training and Research Hospital. Clinical and laboratory data of obese adults who presented to the outpatient clinics between 1 January 2020 and 31 December 2022 were retrieved from the hospital information management system.

The study protocol was approved by the institutional Ethics Committee (Approval No: 345, Date: 3 March 2023). As anonymized, routinely collected clinical data were used, the requirement for written informed consent was waived. All diagnostic cut-offs for prediabetes and diabetes followed American Diabetes Association (ADA) recommendations.

### 2.2. Participants

#### 2.2.1. Inclusion Criteria

Participants were eligible if they met the following conditions: Age ≥ 18 years, obesity: BMI > 30 kg/m^2^, prediabetes (Fasting plasma glucose (FPG) 100–125 mg/dL, and/or HbA1c 5.7–6.4%), underwent a 75 g oral glucose tolerance test (OGTT) as part of routine clinical evaluation, and had available data on key demographic, anthropometric, and laboratory parameters.

#### 2.2.2. Exclusion Criteria

To avoid confounding factors affecting HbA1c, glucose metabolism, or red cell indices, the following individuals were excluded: Age < 18 years, pregnant women, diagnosed diabetes mellitus (Type 1 or Type 2) prior to presentation, use of medications affecting glycemia (glucose-lowering agents [insulin, metformin, alpha-glucosidase inhibitors], glucose-raising medications [systemic corticosteroids, IV dextrose]), known chronic comorbidities (heart failure, chronic kidney disease (eGFR < 60 mL/min/1.73 m^2^), chronic liver disease, cirrhosis, or malabsorption syndromes, coronary artery disease, active malignancy, psychiatric or neurological disorders), history of gastrectomy or bariatric surgery, hemoglobin < 10 g/dL, to avoid anemia-related HbA1c bias, hemoglobinopathy suspicion (samples with “variant flags’’ on HPLC chromatograms), and documented HIV infection.

### 2.3. Sampling Method

This study did not include randomly selected individuals from the general population. Instead, it consisted of obese patients undergoing diagnostic evaluation for dysglycemia in a tertiary care center. All eligible cases within the study dates were consecutively included.

### 2.4. Data Collection

#### Clinical and Demographic Variables

Electronic medical records were reviewed for age, sex, height (cm), weight (kg), BMI (kg/m^2^), waist and hip circumference (cm), systolic and diastolic blood pressure (mmHg), comorbidities and medication history. Blood pressure was measured after 10 min of seated rest, with two readings taken ≥ 2 min apart; the mean value was recorded.

### 2.5. OGTT Procedure

All patients underwent a standard 75 g OGTT performed after a 3-day diet containing ~150 g/day carbohydrates and a 12 h overnight fast. Venous blood samples were collected at: 0 min (fasting plasma glucose), 120 min (post-load plasma glucose).

Samples were transported to the laboratory immediately and analyzed within 30 min according to routine institutional procedures. As the study was retrospective, adherence to the pre-test carbohydrate diet could not be individually verified.

Based on OGTT results, patients were categorized as: isolated impaired fasting glucose (IFG), combined IFG + impaired glucose tolerance (IGT), overt diabetes mellitus (T2DM). Each participant underwent a single OGTT for glycemic classification; repeat confirmatory testing was not available due to the retrospective design.

### 2.6. Laboratory Measurements

All laboratory tests were performed in a single accredited biochemistry laboratory.

HbA1c (%): measured by high-performance liquid chromatography (HPLC) using the Arkray Adams HA-8180V (Arkray Inc., Kyoto, Japan). NGSP-certified and IFCC-traceable Intra-assay CV: <2.0%.

Plasma glucose (mg/dL): hexokinase enzymatic method.

LDL cholesterol (mg/dL): calculated using standard enzymatic colorimetric assays (laboratory-validated protocol).

TSH (µIU/mL): measured by chemiluminescence immunoassay.

Blood pressure was measured in accordance with current hypertension guideline recommendations. After 10 min of seated rest, two measurements were obtained at least 2 min apart using a calibrated automatic sphygmomanometer, and the average value was recorded.

Hypertension was defined as systolic blood pressure ≥ 140 mmHg and/or diastolic blood pressure ≥ 90 mmHg on at least two separate measurements, based on the European Society of Cardiology/European Society of Hypertension [12].

### 2.7. Obesity Classification

Patients were categorized according to WHO BMI criteria:

- Mild obesity: 30–34.9 kg/m^2^
- Moderate obesity: 35–39.9 kg/m^2^
- Morbid obesity: 40–49.9 kg/m^2^
- Super obesity: ≥50 kg/m^2^

Obesity categories were defined according to the World Health Organization classification.

Comparisons of demographic and biochemical variables were performed across obesity categories and across OGTT-defined glycemic subgroups.

**Statistical Analysis:** SPSS version 22.0 for Windows (SPSS Inc., Chicago, IL, USA) was used for statistical analysis. The normality of the data was assessed using the Shapiro–Wilk test. Variables that showed a normal distribution were presented as mean and standard deviation. For variables with a normal distribution, comparisons between two groups were performed using Student’s *t*-test, and comparisons among three groups were performed using the ANOVA test. Variables that did not show a normal distribution were presented as median and interquartile range. For skewed variables, comparisons between two groups were conducted using the Mann–Whitney U test, and comparisons among three groups were performed using the Kruskal–Wallis test. Categorical variables were presented as proportions and percentages of the total, and the Chi-square test was used for comparisons. Spearman’s rank correlation coefficients were calculated to evaluate correlations between variables, as several parameters were not normally distributed. The ability of HbA1c to identify T2DM patients diagnosed using OGTT was tested using Receiver Operating Characteristic (ROC)-Area Under the Curve (AUC) analysis. Exploratory ROC analysis was performed to evaluate the diagnostic performance of HbA1c and to derive a data-driven cut-off using the Youden index. A *p*-value of <0.05 was considered statistically significant. The optimal HbA1c cut-off was determined using Youden’s index. To internally validate the discrimination performance and correct for potential optimism, we applied bootstrap resampling with 1000 iterations and reported the bootstrap optimism-corrected AUC. Because this was a retrospective convenience sample including all eligible patients with complete data, no a priori sample-size or power calculation was performed. To convey precision, exact (Clopper–Pearson) 95% confidence intervals were calculated for sensitivity, specificity, positive predictive value and negative predictive value. The ROC curve of HbA1c for diabetes diagnosis is presented.

This study was not designed as a risk prediction model development study; therefore, multivariable regression modeling was not performed for the primary outcome.

**Figure 1:**
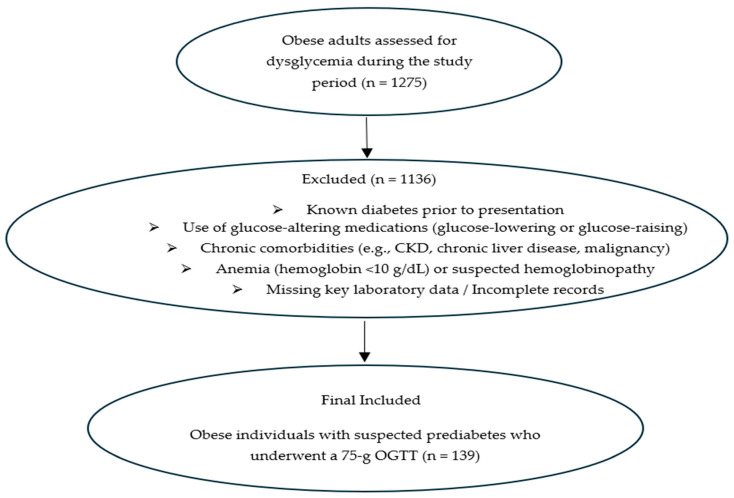
Flow diagram of patient selection and inclusion.

A flow diagram illustrating screening, exclusion criteria, and final inclusion of obese individuals referred for evaluation of suspected prediabetes is presented in Figure 1.

## 3. Results

Demographic and Clinical Characteristics: This study was conducted by examining the data of 139 prediabetic obese patients. Of these, 82.7% (*n* = 115) were female and 17.3% (*n* = 24) were male. The overall mean age was 45.0 ± 12.04 years. The mean age of female patients was 45.18 ± 11.74 years, while that of male patients was 44.13 ± 11.61 years. The median BMI was 39.15 (9.90), with values of 40.06 (9.60) in females and 36.5 (6.70) in males. Detailed sex-stratified demographic characteristics and BMI distributions of the patients are presented in Supplementary Table S1.

In females, BMI was significantly higher (*p* = 0.04), whereas waist circumference was greater in males (*p* = 0.006). No significant differences were observed between male and female participants regarding age, hip circumference, blood pressure values, fasting blood glucose, HbA1c, LDL, or TSH levels (*p* > 0.05).

The mean waist and hip circumference, blood pressure values, fasting blood glucose, HbA1c, LDL, and TSH levels stratified by gender are presented in Supplementary Table S2.

**Figure 2:**
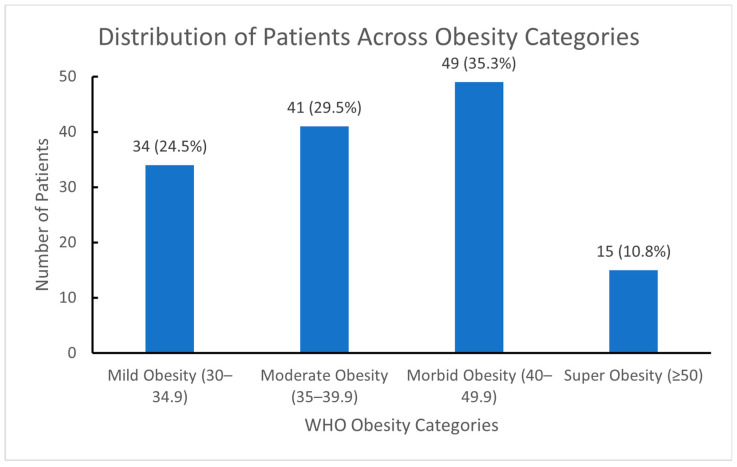
Number and Percentages of Patients Based on BMI. Abbreviations: BMI: Body Mass Index, WHO: World Health Organization.

The percentages and frequencies of patients based on obesity classification are presented in Figure 2.

The mean age of all patients was 45 years, and the mean HbA1c level was 6%. The mean waist circumference was 114.5 cm, while the mean hip circumference was 129.05 cm. The average blood pressure was 126.8/81.1 mmHg (systolic/diastolic). The mean fasting plasma glucose (FPG) level was 109.09 mg/dL, and the mean TSH and LDL levels were 2.22 mIU/L and 121.5 mg/dL, respectively.

The demographic, clinical, and laboratory characteristics of the participants according to BMI categories are presented in Table 1.

An increase in waist circumference (*p* = 0.013), hip circumference (*p* < 0.001), systolic blood pressure (*p* = 0.01), and diastolic blood pressure (*p* = 0.013) was found to be statistically significant with increasing BMI. On the other hand, no significant difference was observed in terms of age, HbA1c, fasting blood glucose, LDL, and TSH levels as obesity severity increased (*p* > 0.05).

Among the 139 patients included in the study, hypertension was detected in 8 patients (23.52%) in the mild obesity group, 4 patients (9.75%) in the moderate obesity group, 8 patients (16.32%) in the morbid obesity group, and 8 patients (53.33%) in the super obesity group, making a total of 28 patients (20.14%) (Table 1).

According to the results of the 75 g OGTT, 37.4% (*n* = 52) of the participants were diagnosed with isolated impaired fasting glucose (IFG), 45.3% (*n* = 63) had combined IFG and impaired glucose tolerance (IGT), and 17.3% (*n* = 24) were diagnosed with overt diabetes mellitus (T2DM). Overall, 17.3% (95% CI: 11.4–24.8%) of participants were diagnosed with diabetes based on OGTT. The mean age of the 139 patients included in the study was 45.0 ± 12.04 years, while the mean age was 44.42 ± 10.96 years in those with IFG, 45.00 ± 12.40 years in those with IFG + IGT, and 46.25 ± 13.64 years in those with overt T2DM.

The demographic, clinical, and baseline laboratory characteristics of the participants according to OGTT categories are presented in Table 2. Anthropometric measurements, including waist and hip circumference, were re-evaluated and confirmed to be correctly recorded.

HbA1c levels of the participants showed a weak–moderate positive correlation with fasting blood glucose (Spearman’s rho = 0.263, *p* = 0.002). However, no significant correlation was found between HbA1c levels and age, BMI, waist and hip circumference, or blood pressure values (*p* > 0.05).

**Figure 3:**
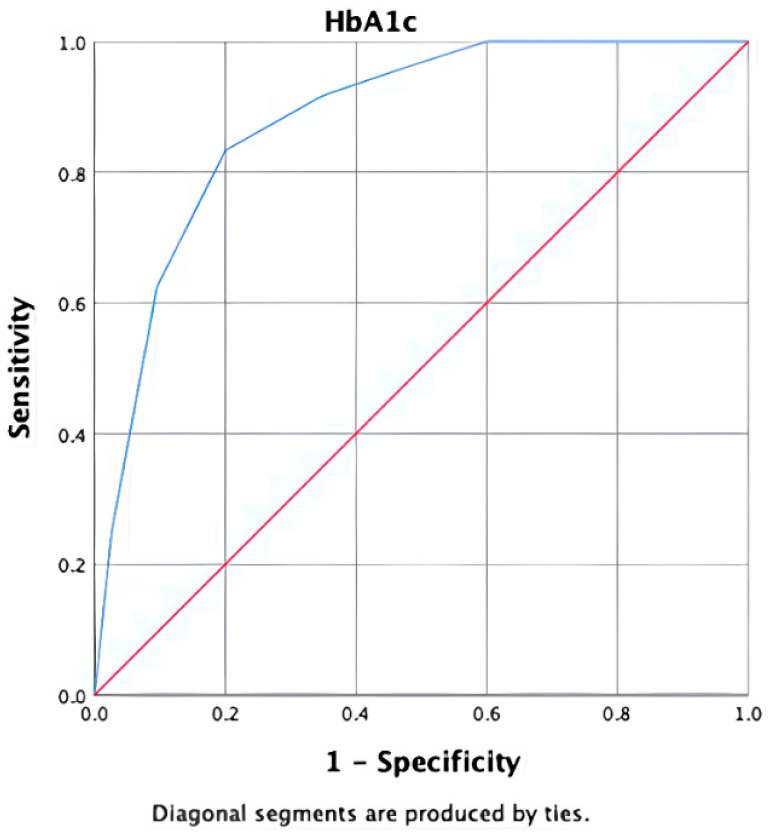
Receiver operating characteristic (ROC) curve of HbA1c for detecting OGTT-defined type 2 diabetes mellitus. The area under the curve (AUC) (Red line) was 0.881 (95% CI: 0.816–0.946). The diagonal line (blue line) represents no discriminative ability.

In the ROC-AUC analysis, the discriminative power of HbA1c in identifying T2DM patients diagnosed using the 75 g OGTT was found to be statistically significant (AUC: 0.881, 95% CI: 0.816–0.946, *p* < 0.001). At this threshold, the positive likelihood ratio (LR+) was 4.17 and the negative likelihood ratio (LR−) was 0.21, indicating moderate rule-in and strong rule-out performance (Figure 3).

Based on the ROC analysis, the cut-off value for HbA1c was determined to be 6.15%. At this threshold, the sensitivity was 83.3% and the specificity was 80% (Table 3). The positive predictive value was calculated as 46.1%, while the negative predictive value was 95.8%. Exact (Clopper–Pearson) 95% confidence intervals were as follows: sensitivity 83.3% (95% CI: 62.6–95.3), specificity 80.0% (95% CI: 71.5–86.9), PPV 46.5% (95% CI: 31.2–62.3), and NPV 95.8% (95% CI: 89.7–98.9). The prevalence of OGTT-diagnosed diabetes in the cohort was 17.3% (24/139) (95% CI: 11.4–24.6). Bootstrap internal validation (1000 iterations) yielded a similar optimism-corrected AUC, supporting the robustness of the discrimination performance.

These results indicate that an HbA1c cut-off of 6.15% is associated with the observed sensitivity, specificity, and negative predictive value for identifying overt diabetes in obese individuals.

## 4. Discussion

In this study, we evaluated the diagnostic performance between OGTT and HbA1c in diagnosing diabetes among individuals with obesity. Our primary finding was that the HbA1c value showing the best diagnostic performance for detecting OGTT-defined diabetes in obese individuals with suspected prediabetes was 6.15%, which is lower than the universally accepted 6.5% cut-off recommended by current guidelines [5]. Additionally, increasing BMI was associated with higher systolic and diastolic blood pressure values, and 17.3% of individuals classified as prediabetic based on HbA1c were diagnosed with overt diabetes using the 75 g OGTT. These results suggest that HbA1c may underestimate glycemic burden in obese patients and highlight the need for obesity-specific screening considerations rather than revised population-wide diagnostic thresholds.

Several obesity-related physiological mechanisms may explain why the optimal HbA1c threshold was lower in our cohort. Obesity is characterized by chronic low-grade inflammation and increased oxidative stress, both of which shorten erythrocyte lifespan. Because HbA1c reflects cumulative glycation over the 120-day erythrocyte cycle, a reduced erythrocyte lifespan leads to artificially lower HbA1c values for the same level of glycemia. In addition, obesity is associated with altered iron metabolism, elevated hepcidin levels, and a higher prevalence of subclinical functional iron deficiency, all of which can modify glycation kinetics and lower HbA1c independently of glucose. These mechanisms collectively support the possibility that the standard 6.5% HbA1c threshold may underestimate true glycemic burden in obese individuals [8].

The observed increase in blood pressure with higher BMI in our study can be explained by the well-established pathophysiological pathways linking obesity to hypertension. Excess adiposity increases sympathetic nervous system activity, enhances activation of the renin–angiotensin–aldosterone system, and promotes sodium retention, all of which elevate blood pressure. Adipose tissue also acts as an endocrine organ, secreting pro-inflammatory cytokines and adipokines such as leptin, which further stimulate sympathetic outflow and impair endothelial nitric oxide production. These mechanisms contribute to increased arterial stiffness and vascular resistance, providing a biological explanation for the higher blood pressure values observed with increasing BMI [12].

The demographic and anthropometric characteristics of our cohort were generally consistent with previously published studies conducted in obese populations undergoing evaluation for dysglycemia. Similar to prior reports, obesity severity was associated with higher blood pressure values, while HbA1c levels did not differ substantially across BMI categories. These findings suggest that, within obese individuals referred for metabolic evaluation, HbA1c may not adequately reflect the full spectrum of glucose intolerance, supporting the continued role of OGTT in this high-risk group.

In our study, hypertension prevalence increased with obesity severity and was highest in the super-obese group. In addition, waist and hip circumference, as well as systolic and diastolic blood pressure values, showed a statistically significant increase with rising BMI, supporting the known association between adiposity and blood pressure elevation.

Previous studies have consistently demonstrated that reliance on fasting glucose or HbA1c alone may lead to underdiagnosis of diabetes in high-risk obese populations. Several reports have shown that a substantial proportion of individuals classified as prediabetic are subsequently diagnosed with overt diabetes following OGTT. Consistent with these findings, 17.3% of obese individuals in our cohort, all of whom were classified as prediabetic based on HbA1c, were diagnosed with overt diabetes by OGTT [13,14]. This observation underscores the limited diagnostic performance of HbA1c alone in this setting and highlights the continued importance of OGTT for accurate detection of dysglycemia in obese individuals.

Previous studies have reported a weak to moderate correlation between fasting blood glucose and HbA1c, particularly in obese populations, reflecting the complex relationship between chronic and short-term glycemic exposure [15]. In line with these observations, HbA1c values in our study showed a weak–moderate positive correlation with fasting glucose (Spearman’s rho = 0.263, *p* = 0.002). Although the magnitude of this association was modest, its statistical significance supports the physiological plausibility of using HbA1c as a screening marker in obese individuals. The absence of a stronger correlation may be explained by obesity-related factors affecting hemoglobin glycation kinetics. Given our sample size, subtle BMI-stratified differences could not be reliably detected, and larger studies are warranted to further explore BMI-dependent variations in HbA1c performance.

Although obesity is strongly associated with the development of type 2 diabetes, the relationship between BMI and HbA1c appears to be heterogeneous, supporting the need for confirmatory testing such as OGTT in obese individuals.

In our study, hypertension was detected in 20.14% of obese patients. A weak–moderate positive correlation was observed between HbA1c levels and fasting blood glucose.

According to the 2024 American Diabetes Association (ADA) and TEMD guidelines, diabetes is diagnosed when HbA1c ≥ 6.5% [5,16]. Given the well-established association between obesity and dysglycemia, obese individuals frequently undergo further evaluation when glycemic abnormalities are suspected. In this context, our study focused on obese patients referred for assessment of prediabetes and evaluated the diagnostic performance of HbA1c against OGTT-defined diabetes. In our cohort, the ROC–AUC analysis demonstrated that HbA1c had significant discriminatory ability for detecting OGTT-diagnosed diabetes (AUC: 0.881, 95% CI: 0.816–0.946, *p* < 0.001). An HbA1c threshold of 6.15% showed a sensitivity of 83.3% and a specificity of 80%, with a high negative predictive value (95.8%). However, because our study population consisted of obese individuals already undergoing evaluation in a tertiary care setting, these findings should be interpreted within the framework of a diagnostic accuracy study rather than as evidence for redefining population-wide diagnostic thresholds. Our results suggest that, in obese individuals with suspected prediabetes, HbA1c may serve as a useful screening parameter to identify patients who warrant confirmatory OGTT testing.

These findings suggest that in obese populations, the conventional HbA1c threshold of 6.5% may underestimate the true prevalence of diabetes. Identifying an optimized cut-off—such as the 6.15% value determined in our study—may improve diagnostic sensitivity and facilitate earlier detection of dysglycemia in high-risk obese individuals. Incorporating obesity-specific HbA1c thresholds into clinical screening strategies could enhance diagnostic accuracy and help prevent delays in diabetes diagnosis.

### Limitations and Strengths

The scarcity of similar studies and data in the literature highlights the significance of our study in contributing to existing knowledge. Further large-scale, multicenter investigations with larger cohorts are needed to validate and expand our findings before any implications can be considered for clinical guidelines. In addition, the relatively limited sample size of our cohort may have reduced the precision of the estimated sensitivity and specificity values, which should be considered when interpreting the diagnostic accuracy results.

From a clinical perspective, our findings do not support replacing OGTT with HbA1c for the diagnosis of diabetes in obese individuals. Instead, HbA1c appears to function as a useful triage tool to identify obese patients with suspected prediabetes who warrant confirmatory OGTT testing, particularly given its strong negative predictive value.

Although our study is original and clinically relevant, its retrospective design precluded verification of adherence to the recommended 3-day high-carbohydrate diet prior to the OGTT. Moreover, because our cohort consisted exclusively of obese individuals already classified as prediabetic, comparison with a normoglycemic reference group was not possible, which limits assessment of diagnostic discordance metrics such as kappa for prediabetes classification. While a multivariable logistic regression model adjusted for age, sex, or BMI could have provided additional analytical depth, it could not be implemented due to the study’s limited sample size and retrospective design, as the primary objective focused on diagnostic accuracy rather than multivariable prediction modeling. Rather than proposing a new diagnostic threshold for diabetes, our findings suggest that HbA1c may serve as a triage tool to identify obese individuals with suspected prediabetes who warrant confirmatory OGTT testing. Because our cohort consisted of obese individuals referred for dysglycemia evaluation, the identified HbA1c cut-off should not be generalized to the entire obese population. In line with STARD recommendations, these sources of spectrum bias, incorporation bias, and cut-point optimism should be considered when interpreting the diagnostic performance estimates.

Rather than proposing a new diagnostic threshold for diabetes, our findings suggest that HbA1c may serve as a practical triage tool to identify obese individuals with suspected prediabetes who warrant confirmatory OGTT testing. In this high-risk population, reliance on HbA1c alone may lead to missed diagnoses, whereas a triage-based approach may help optimize OGTT utilization in clinical practice.

Future studies should focus on prospective, multicenter diagnostic accuracy designs including obese individuals across the full glycemic spectrum, from normoglycemia to overt diabetes. External validation of data-derived HbA1c cut-offs and evaluation of sex-specific and obesity class–specific performance are also needed before any consideration of broader clinical implementation.

## 5. Conclusions

In obese individuals referred for evaluation of suspected prediabetes, an HbA1c value of 6.15% demonstrated the best performance for detecting OGTT-defined diabetes. This finding should not be interpreted as a population-wide diagnostic threshold, but rather as a screening aid to guide selective use of OGTT in high-risk obese patients. Given the selected nature of the cohort and the retrospective design, further prospective studies including normoglycemic and diabetic obese individuals are required before any modification of diagnostic criteria can be considered.

## Figures and Tables

**Table 1:** Demographic, Clinical, and Laboratory Characteristics of Participants According to BMI Categories.

	Total (*n* = 139)	Mild Obesity (*n* = 34)	Moderate Obesity (*n* = 41)	Morbid Obesity (*n* = 49)	Super Obesity (*n* = 15)	*p*-Value
Age, Mean (SD)	45 (12.03)	46.18 (12.4)	42.68 (11.7)	43.63 (11.2)	53.13 (11.8)	0.12
HbA1c (%), Mean (SD)	6.0 (0.22)	6.0 (0.22)	6.01 (0.22)	5.99 (0.23)	6.03 (0.19)	0.95
Waist Circumference (cm), Mean (SD)	114.5 (11.1)	105.4 (6.9)	109.8 (7.2)	119.6 (9.0)	131.2 (4.25)	0.013
Hip Circumference (cm), Mean (SD)	129.05 (14.7)	117.6 (5.3)	122.4 (5.9)	132.8 (8.7)	160.6 (12.4)	<0.001
SBP (mmHg), Mean (SD)	126.8 (13.2)	126.3 (12.9)	124.3 (13.4)	125.9 (11.8)	137.8 (13.4)	0.01
DBP (mmHg), Mean (SD)	81.12 (7.6)	79.4 (7.8)	80.1 (8.5)	81.6 (7.1)	85.8 (4.2)	0.013
FBG (mg/dL), Mean (SD)	109.09 (7.06)	109.29 (7.3)	109.34 (6.8)	108.22 (6.9)	110.80 (7.8)	0.66
LDL (mg/dL), Mean (SD)	121.5 (33.8)	121.88 (24.9)	118.98 (37.0)	127.02 (37.6)	110.32 (27.8)	0.18
TSH (mIU/L),	2.22 (1.56)	2.02 (1.34)	2.15 (1.71)	2.25 (1.37)	2.78 (2.11)	0.45
Hypertension, *n* (%)	28 (20.1%)	8 (23.5%)	4 (9.7%)	8 (16.3%)	8 (53.3%)	0.003

Abbreviations: *n*: Number, SD: Standard Deviation, BMI: Body Mass Index, SBP: Systolic Blood Pressure, DBP: Diastolic Blood Pressure, FBG: Fasting Blood Glucose, TSH: Thyroid Stimulating Hormone. Values are presented as mean ± SD or median (IQR) as appropriate. *p*-values were obtained using Student’s *t*-test or ANOVA for normally distributed variables, and the Mann–Whitney U test or Kruskal–Wallis test for non-normally distributed variables, as applicable. *p*-values for categorical variables were calculated using the chi-square test. *p*-values for continuous variables were calculated using one-way ANOVA.

**Table 2:** Demographic, Clinical, and Laboratory Characteristics of Participants According to OGTT Categories.

Parameter	IFG (*n* = 52)	IFG + IGT (*n* = 63)	Overt T2DM (*n* = 24)	*p*-Value
Age Mean (SD)	44.42 (10.96)	45.00 (12.40)	46.25 (13.64)	0.830
BMI (kg/m^2^), Median (IQR)	39.15 (11.4)	39.30 (10.0)	38.73 (9.8)	0.636
Waist Circumference (cm), Median (IQR)	111.5 (19)	112 (18)	111.5 (14)	0.769
Hip Circumference (cm), Median (IQR)	112.5 (19)	128 (16)	122 (12)	0.113
SBP (mmHg), Median (IQR)	120.5 (13)	122 (21)	125.5 (17)	0.120
DBP (mmHg), Median (IQR)	80.5 (8)	80.0 (12)	81.5 (10)	0.290
FBG (mg/dL), Median (IQR)	105 (9)	109 (13)	113.5 (14)	<0.001
HbA1c (%), Median (IQR)	5.85 (0.4)	6.00 (0.4)	6.30 (0.2)	<0.001
LDL (mg/dL), Median (IQR)	114.5 (52)	119 (44)	133 (45)	0.372
TSH (mIU/L), Median (IQR)	1.78 (2)	1.83 (1)	1.65 (1)	0.270

Abbreviations: *n*: Number of patients, SD: Standard deviation, IQR: Interquartile range, BMI: Body mass index, SBP: Systolic blood pressure, DBP: Diastolic blood pressure, FBG: Fasting blood glucose, TSH: Thyroid-stimulating hormone, IFG: Impaired fasting glucose, IGT: Impaired glucose tolerance, T2DM: type 2 Diabetes mellitus. All waist and hip circumference measurements were obtained from electronic medical records and were checked for accuracy. Values are presented as median (interquartile range) due to non-normal distribution.

**Table 3:** Diagnostic Performance of HbA1c > 6.15% for Detecting Overt Diabetes Based on OGTT.

OGTT Result	Overt T2DM	No T2DM	Total
HbA1c > 6.15%, *n* (%)	20 (14.4)	23 (16.5)	43 (30.9)
HbA1c < 6.15%, *n* (%)	4 (2.9)	92 (11.4)	96 (69.1)
Total, *n* (%)	24 (17.3)	115 (82.7)	

Abbreviations: *n*: Number of patients, OGTT: Oral Glucose Tolerance Test, T2DM: type 2 Diabetes Mellitus.

## Data Availability

All data can be made available by the corresponding author upon request.

## Supplementary Materials

The following supporting information can be downloaded at: https://www.mdpi.com/article/10.3390/jcm15010374/s1, Table S1: Sex-Stratified Demographic Characteristics and BMI-Based Patient Distribution; Table S2: Sex-Stratified Anthropometric, Clinical, and Laboratory Characteristics of Participants.
