# Supplementary material for: Diagnostic Performance of HbA1c for Detecting OGTT-Diagnosed Diabetes in Obese Individuals with Suspected Prediabetes
Source: J Clin Med. 2026 Jan 4;15(1):374. doi: 10.3390/jcm15010374 (PMC12786714; doi:10.3390/jcm15010374)
Supplement: Supplementary file 1 [file jcm-15-00374-s001.zip › jcm-4061245-supplementary.pdf]

Supplementary Table S1: Sex-Stratified Demographic Characteristics and BMI-Based Patient Distribution

	Total (n=139)	Female (n=115)	Male (n=24)
Number, n (%)	139	115 (82.7)	24 (17.3)
Mean Age	45.00	45.18	44.13
BMI (kg/m^2), Median (IQR)	39.15 (9.90)	40.06 (9.60)	36.50 (6.70)
Mild Obesity, n (%)	34	24 (70.6)	10 (29.4)
Moderate Obesity, n (%)	41	32 (78.1)	9 (21.9)
Morbid Obesity, n (%)	49	47 (95.9)	2 (4.1)
Super Obesity, n (%)	15	12 (80)	3 (20)
Impaired Fasting Glucose (IFG), n (%)	52	45 (86.5)	7 (13.5)
IFG + Impaired Glucose Tolerance (IGT), n (%)	63	51 (80.9)	12 (19.1)
Overt Diabetes Mellitus (DM), n (%)	24	19 (79.1)	5 (20.9)

Abbreviations: n: number, SD: standard deviation, IQR: interquartile range, BMI: body mass index, IFG: impaired fasting glucose, IGT: impaired glucose tolerance, DM: diabetes mellitus, OGTT: oral glucose tolerance test.

Supplementary Table S2: Sex-Stratified Anthropometric, Clinical, and Laboratory Characteristics of Participants

	Total (n=139)	Female (n=115)	Male (n=24)	p-value
Age, Mean (SD)	45.00 (12.04)	45.18 (11.74)	44.13 (11.61)	0.697
BMI (kg/m²), Median (IQR)	39.15 (9.90)	40.06 (9.60)	36.50 (6.70)	**0.04**
Waist Circumference (cm), Median (IQR)	112 (17)	110 (17)	116 (17)	**0.006**
Hip Circumference (cm), Median (IQR)	125 (17)	125 (17)	125 (13)	0.487
SBP (mmHg), Median (IQR)	122 (15)	122 (15)	124 (20)	0.374
DBP (mmHg), Median (IQR)	80 (8)	80 (7)	81 (10)	0.394
Fasting Blood Glucose (mg/dL), Median (IQR)	108 (11)	108 (10)	111 (14)	0.164
HbA1c (%), Median (IQR)	6.0 (0.4)	6.0 (0.4)	6.0 (0.4)	0.635
LDL (mg/dL), Median (IQR)	120 (45)	120 (45)	123 (57)	0.44
TSH (IU/L),	1.80 (1.00)	1.76 (1.00)	1.90 (1.00)	0.80

Abbreviations; n: number, SD: standard deviation, IQR: interquartile range, BMI: body mass index, SBP: systolic blood pressure, DBP: diastolic blood pressure, FBG: fasting blood glucose, TSH: thyroid-stimulating hormone. Values are presented as mean ± SD or median (IQR) as appropriate. p-values were obtained using Student's t-test or ANOVA for normally distributed variables, and Mann–Whitney U test or Kruskal–Wallis test for non-normally distributed variables, as applicable
